# Importance of the Walden Inversion for the Activity Volcano Plot of Oxygen Evolution

**DOI:** 10.1002/advs.202305505

**Published:** 2023-10-30

**Authors:** Kai S. Exner

**Affiliations:** ^1^ Faculty of Chemistry Theoretical Inorganic Chemistry University Duisburg‐Essen Universitätsstraße 5 45141 Essen Germany; ^2^ Cluster of Excellence RESOLV 44801 Bochum Germany; ^3^ Center for Nanointegration (CENIDE) Duisburg‐Essen 47057 Duisburg Germany

**Keywords:** descriptor approach, OER, reaction mechanism, volcano plot, Walden inversion

## Abstract

Since the birth of the computational hydrogen electrode approach, it is considered that activity trends of electrocatalysts in a homologous series can be quantified by the construction of volcano plots. This method aims to steer materials discovery by the identification of catalysts with an improved reaction kinetics, though evaluated by means of thermodynamic descriptors. The conventional approach for the volcano plot of the oxygen evolution reaction (OER) relies on the assumption of the mononuclear mechanism, comprising the ^*^OH, ^*^O, and ^*^OOH intermediates. In the present manuscript, two new mechanistic pathways, comprising the idea of the Walden inversion in that bond‐breaking and bond‐making occurs simultaneously, are factored into a potential‐dependent OER activity volcano plot. Surprisingly, it turns out that the Walden inversion plays an important role since the activity volcano is governed by mechanistic pathways comprising Walden steps rather than by the traditionally assumed reaction mechanisms under typical OER conditions.

## Graphical abstract

The activity volcano plot for oxygen evolution is governed by mechanistic pathways consisting of Walden inversion steps.

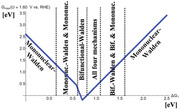

## 1 Introduction

Due to the rapid depletion of fossil‐based energy sources, global warming, and the increasing world population, there is an urgent need of new concepts for energy storage and conversion to meet the demand of our everyday lives.^[^ ^1^ ^]^ One of our main hopes is dedicated to gaseous hydrogen, a clean fuel with high gravimetric energy density, which can be produced almost without CO_2_ emissions by the electrochemical water splitting if the required electricity is supplied by renewables.^[^ ^2^, ^3^, ^4^ ^]^ The water‐splitting electrolysis consists of two separated processes, namely the hydrogen evolution (HER) and oxygen evolution (OER) reactions at the cathode and anode, respectively.^[^ ^5^ ^]^ While the HER, 2 H^+^ + 2 e^–^ → H_2_, *U* ^0^_HER_ = 0 V versus reversible hydrogen electrode (RHE), is a facile two‐electron process with negligible losses relating to the required overpotential, the OER, 2 H_2_O → O_2_ + 4 H^+^ + 4 e^–^, *U* ^0^_OER_ = 1.23 V versus RHE, represents the bottleneck in acidic and alkaline electrolyzers due to its slow reaction kinetics, accompanied with significant overpotentials to reach current densities of practical interest. Therefore, in the realm of the energy transition, tremendous efforts have been made to search for materials that are capable of catalyzing the OER more efficiently ^[^ ^6^ ^]^; yet, hitherto, with little success considering that scarce noble metal‐based catalysts based on IrO_2_ or RuO_2_ coatings are the only relevant electrocatalysts for the acidic water splitting due to stability reasons.^[^ ^7^, ^8^ ^]^

The search for OER catalysts has largely been driven by electronic structure calculations in the density functional theory approximation, which can be related to the introduction of the computational hydrogen electrode (CHE) approach by Nørskov and coworkers in 2004.^[^ ^9^ ^]^ This framework allows computing the free‐energy changes, Δ*G*_j_, of mechanistic pathways at electrified solid/ liquid interfaces as encountered during the electrolysis of water. The simplicity of this method has spurred its success in the community of computational scientists ^[^ ^10^ ^]^ while meanwhile, even experimentalists have adopted this mindset by extracting (thermodynamic) free‐energy changes from (kinetic) cyclic voltammetry measurements.^[^ ^11^, ^12^ ^]^

Materials screening by the CHE approach is facilitated by the coupling of the derived Δ*G*_j_ values with the Sabatier principle ^[^ ^13^ ^]^ and the Brønsted–Evans–Polanyi (BEP) relation ^[^ ^14^ ^]^ to discuss activity trends in the framework of the thermodynamic overpotential, *η*_TD_, an activity measure that refers to the largest free‐energy change under equilibrium conditions. The fate of this approach is that the quality of the theoretical predictions significantly depends on the assumed mechanistic pathway.^[^ ^15^, ^16^, ^17^, ^18^ ^]^ Since the early works of Rossmeisl and coworkers on the OER over transition‐metal oxides, it has been considered a paradigm that the OER is described by the so‐called mononuclear description, consisting of the ^*^OH, ^*^O, and ^*^OOH adsorbates (cf. Equations (1)–(4)) ^[^ ^19^ ^]^:

(1) $$M+H_{2}O\;\;\to M-\mathrm{OH}+H^{+}+e^{-}\;\;\;\;\Delta G_{1}$$

(2) $$M-\mathrm{OH}\;\;\;\to M-O+H^{+}+e^{-}\;\;\;\;\Delta G_{2}$$

(3) $$M-O+H_{2}O\;\to M-\mathrm{OOH}+H^{+}+e^{-}\;\Delta G_{3}$$

(4) $$M-\mathrm{OOH}\;\;\;\;\to M+O_{2(g)}+H^{+}+e^{-}\;\;\;\Delta G_{4}$$

In Equations (1)–(4), M denotes the catalytically active surface site (e.g., an undercoordinated metal atom), and the four OER free‐energy changes meet the criterion of Equation (5) following the notion of gas‐phase error corrections ^[^ ^20^, ^21^ ^]^:

(5) $$\Delta G_{1}+\Delta G_{2}+\Delta G_{3}+\Delta G_{4}=+4.92\mathrm{eV}\;@U=0\;V\;\mathrm{vs}.\;\mathrm{RHE}$$

The thermodynamic overpotential ^[^ ^9^ ^]^ serving as the activity descriptor is given by Equation (6):

(6) $$\begin{array}{ccc} \eta_{\mathrm{TD}} & = & \max\{\Delta G_{1}-1.23\;\mathrm{eV};\;\Delta G_{2}-1.23\;\mathrm{eV};\\ & & \Delta G_{3}-1.23\;\mathrm{eV};\;\Delta G_{4}-1.23\;\mathrm{eV}\}/e \end{array}$$

Plotting the thermodynamic overpotential as a function of the free‐energy change Δ*G*_1_ or Δ*G*_2_ in a class of materials gives rise to the construction of an activity volcano plot,^[^ ^22^ ^]^ which is used to comprehend activity trends and to steer the search for material motifs with higher intrinsic OER activity. The corresponding activity volcano for the mononuclear pathway (cf. Equations (1)–(4)) is shown in Figure S1 (Supporting Information), indicating that either ^*^O formation or ^*^OOH formation reveals the largest free‐energy change under equilibrium conditions, and thus, is reconciled with the potential‐determining step (PDS).^[^ ^23^ ^]^ Using the tacit assumption that the potential‐ and rate‐determining (RDS) steps are identical, a supposition, which is not always fulfilled,^[^ ^23^, ^24^ ^]^ the search for material motifs has been governed by stabilizing either the ^*^OOH adsorbate (left leg) or the ^*^O adsorbate (right leg) to obtain higher electrocatalytic activity. Regrettably, the success of this procedure is modest when critically analyzing the progress in the development of OER catalysts, as evident by the overview article of Seh et al.^[^ ^6^ ^]^

In the last years, significant progress has been made relating to the theoretical description of the OER and the concept of volcano plots for the heuristic materials screening.^[^ ^25^, ^26^, ^27^, ^28^, ^29^, ^30^ ^]^ Relating to the latter, it is noteworthy that the approximation of the electrocatalytic activity has been refined by the introduction of an advanced activity descriptor, *G*_max_(*U*), a potential‐dependent activity measure based on the notion of the free‐energy span model.^[^ ^24^, ^31^ ^]^ While the thermodynamic overpotential analyzes the free‐energy changes of a mechanistic pathway only at the equilibrium potential of the OER, *G*_max_(*U*) offers a potential‐dependent description of the electrocatalytic activity, comprising that several steps can govern the rate, and the limiting steps can alter upon enhanced driving force, as encountered in experiments when referring to a change in the Tafel slope. Using the concept of *G*_max_(*U*), volcano plots for the OER have been derived in a recent contribution,^[^ ^32^ ^]^ indicating that the common presumption of the mononuclear description only is too simplistic, at least for highly active electrocatalyst at the volcano apex. There, several pathways can be operative in dependence of the descriptor Δ*G*_1_, see Figures S2 and S3 (Supporting Information) in the Section S1 (Supporting Information). Most notably, a change in the preferred mechanism with increasing overpotential is encountered, underpinning that so far, the modeling of OER catalysts by means of DFT approaches has overlooked the mechanistic complexity of the four proton‐coupled electron transfer steps.^[^ ^32^ ^]^

While the latest work of the author has focused on the different OER mechanisms reported in the literature,^[^ ^19^, ^33^, ^34^, ^35^, ^36^, ^37^ ^]^ it needs to be emphasized that the concept of volcano plots addresses only the activity of electrocatalysts whereas approaches to capture the stability of electrode materials are rare.^[^ ^38^, ^39^ ^]^ In this context, the recent contribution by Hess and Over must be highlighted where the authors unravel the competing dissolution and OER activity of a model RuO_2_(100) surface by means of DFT calculations.^[^ ^40^ ^]^ They report that the degradation of the RuO_2_ electrode is governed by a surface step akin to the Walden inversion, which relates to the simultaneous bond‐breaking and bond‐making including an inversion of the stereochemistry. While activity and stability appear to be coupled in the OER ^[^ ^41^, ^42^ ^]^ albeit a few counterexamples have been reported,^[^ ^43^, ^44^ ^]^ so far, the opportunity of a Walden inversion step relating to OER activity has not been tackled. In the present manuscript, I outline the importance of the Walden inversion for OER activity volcanoes. It is demonstrated that mechanistic pathways comprising a Walden step excel the traditionally assumed mechanisms in terms of predicted electrocatalytic activity. This finding does not only reveal another link between the electrocatalytic activity and stability of electrocatalysts, but also purports a change in the mindset in that the Walden inversion may play an important role in the mechanistic processes of proton–coupled electron transfer steps at electrified solid/ liquid interfaces even beyond the OER.

## 2 Results and Discussion

In the Section S2 (Supporting Information), it Is illustrated how the free‐energy changes Δ*G*_α_ (α = 1, …, 4) of the mononuclear mechanism are related by a rigorous thermodynamic treatment to the free energies of the reaction intermediates. This is only possible by making use of the scaling relations between the ^*^OH and ^*^O as well as the ^*^OH and ^*^OOH adsorbates, as reported in the literature.^[^ ^22^ ^]^ Knowledge of the reaction intermediates’ free energies in dependence of the applied electrode potential enables determining the activity descriptor *G*_max_(*U*),^[^ ^24^, ^31^ ^]^ which is used as measure for the electrocatalytic activity in the volcano plot on the y axis. On the x axis, the free‐energy change Δ*G*_1_ (cf. Equation (1)) is chosen as the descriptor.^[^ ^19^ ^]^ In this contribution, we do not explicitly calculate the Δ*G*_1_ values of various catalysts in a class of materials, but rather make use of a data‐driven methodology as recently introduced by the author.^[^ ^45^ ^]^ Therein, we define a basis set of Δ*G*_1_ values that represent the parameter space of available materials in the OER.^[^ ^32^ ^]^ Given that Rossmeisl and coworkers reported that basically all relevant materials to the oxygen electrocatalysis are within Δ*G*_1_ = [−0.50, 2.50] eV,^[^ ^46^ ^]^ this free‐energy regime with a step size of 0.01 eV is adopted to compile activity volcano plots at four different applied electrode potentials, namely *U* = 1.23, 1.40, 1.60, and 1.80 V versus RHE. All further information relating to the modeling approach is provided in the Supporting Information.

While Equations (1)–(4) indicate the traditional mononuclear mechanism, this mechanistic pathway can be rewritten by the inclusion of a Walden step, which comprises simultaneous bond‐breaking and bond‐making events. As a fundamental consequence, the reference structure in the adjusted mechanism must be changed since in this case, there is no unoccupied active site, M, available due to the simultaneous release of gaseous oxygen and the formation of the ^*^OH adsorbate (cf. Equations (7)–(10)):

(7) $$M-\mathrm{OH}\;\;\to M-O+H^{+}+e^{-}\;\;\;\;\Delta G_{5}$$

(8) $$M-O+H_{2}O\;\;\to M-\mathrm{OOH}+H^{+}+e^{-}\;\;\;\Delta G_{6}$$

(9) $$M-\mathrm{OOH}\;\;\;\to M-\mathrm{OO}+H^{+}+e^{-}\;\;\Delta G_{7}$$

(10) $$M-\mathrm{OO}+H_{2}O\;\to \mathrm{HO}-M+O_{2(g)}+H^{+}+e^{-}\;\Delta G_{8}$$

It should be emphasized that Equation (10) with the free‐energy change Δ*G*_8_ refers to the Walden inversion step as the formation of gaseous oxygen and the adsorption of water take place concurrently, comprising that the reactant (water) enters the active center from a different side than the product (O_2_) leaves the active center. The revised mononuclear mechanism, denoted as mononuclear‐Walden, has two steps (cf. Equations (7)–(8)) in common with the original description of Equations (1)–(4) whereas the last two elementary steps differ from the traditional description.

For the mononuclear‐Walden mechanism, the same thermodynamic analysis as encountered for the mononuclear mechanism of Equations (1)–(4) is executed by relating the free‐energy changes Δ*G*_α_ (α = 5, …, 8) of Equations (7)–(10) via scaling relations to the electrocatalytic activity in the approximation of *G*_max_(*U*). A dedicated derivation of this procedure is provided in Section S3 (Supporting Information).

**Figure 1:**
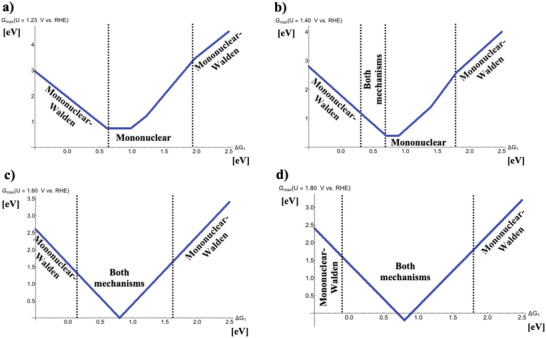
Potential‐dependent volcano plots for the mononuclear and the mononuclear‐Walden pathways of the oxygen evolution reaction at a) *U* = 1.23 V versus RHE, b) *U* = 1.40 V versus RHE, c) *U* = 1.60 V versus RHE, and d) *U* = 1.80 V versus RHE. The energetically favored mechanisms in the approximation of *G*_max_(*U*) as a potential‐dependent activity descriptor are indicated in dependence of the adsorption‐free energy of the ^*^OH intermediate, Δ*G*_1_. To derive the volcano curves, the following scaling relations are considered: Δ*G*_2_ + Δ*G*_3_ = 3.20 eV and Δ*G*_2_ = 2 × Δ*G*_1_.

Knowledge of *G*_max_(*U*) in dependence of the descriptor Δ*G*_1_ for each mechanism gives rise to the construction of a volcano plot in that both mechanistic pathways, namely the traditional mononuclear mechanism and the mononuclear‐Walden mechanism, are plotted in the same diagram at *U* = 1.23 V versus RHE. The corresponding raw data for this practice is provided in Section S4 (Supporting Information) by referring to Figure S4 (Supporting Information), indicating that at different bond strengths of the ^*^OH adsorbate, a different mechanism is energetically preferred. The resulting activity volcano plot is constructed by extracting the segments of the favored mechanistic pathway in dependence of Δ*G*_1_, as summarized in **Figure** **1**. While the conventional method to compile volcano plots based on the notion of the thermodynamic overpotential relies on the assessment of the energetics at *U* = 1.23 V versus RHE (cf. Figure S1, Supporting Information),^[^ ^22^ ^]^ it is noteworthy that the activity measure *G*_max_(*U*) offers a potential‐dependent contemplation of the energetics,^[^ ^32^ ^]^ rendering the construction of volcano plots at any electrode potential *U* > 1.23 V versus RHE possible. Therefore, the OER activity volcano is depicted at four different electrode potentials, namely *U* = 1.23, 1.40, 1.60, and 1.80 V versus RHE, in Figure 1. Please note that for OER volcano plots, Δ*G*_2_ is commonly applied as the descriptor on the x‐axis.^[^ ^6^ ^]^ Due to the scaling between the ^*^OH and ^*^O intermediates, it is possible to translate Δ*G*_1_ to Δ*G*_2_, though this will not impact the derived conclusions. Additionally, the volcano plots shown in this contribution are displayed as inverted volcanoes to underpin that the activity descriptor *G*_max_(*U*) rather than the conventionally applied notion of *η*_TD_ is used as a measure for the electrocatalytic activity.

Figure 1 reveals that under equilibrium conditions, the mononuclear‐Walden mechanism is particularly favored at the legs of the volcano plot, which is governed by inactive OER materials. In contrast, at the top of the volcano the traditional mononuclear pathway is energetically preferred over the mononuclear‐Walden sequence. At first glance, this may indicate that the mononuclear‐Walden mechanism may not be of relevance for the identification of high‐performance electrocatalysts, however, a different situation is encountered if the applied electrode potential is increased by ≈200 to 400 mV: while at *U* = 1.40 V versus RHE, the two mechanistic pathways compete close to the volcano top (0.3 eV < Δ*G*_1_ < 0.7 eV), at *U* = 1.60 V versus RHE, the volcano apex is described by the mononuclear and mononuclear‐Walden mechanisms both (0.15 eV < Δ*G*_1_ < 1.65 eV). This finding does not change if the applied electrode potential is further increased to *U* = 1.80 V versus RHE while quantitatively, the competition between the two pathways is even more pronounced at the volcano legs (–0.10 eV < Δ*G*_1_ < 1.85 eV). For a discussion on the free‐energy spans governing the OER activity volcano, the reader is referred to Section S4 (Supporting Information).

The activity volcano plots of Figure 1 underpin that for inactive OER materials, a change in the reaction mechanism with increasing overpotential is unlikely since the mononuclear‐Walden mechanism only governs the volcano legs. On the contrary, it appears that for highly active OER catalysts, a switch in the reaction mechanism with enhanced driving force is probable since the mononuclear pathway is observed for low overpotentials (cf. Figure 1a,b) whereas for large overpotentials, the mononuclear‐Walden mechanism comes additionally into play (cf. Figure 1c,d). The feature of a changing reaction mechanism with increasing overpotential has been recently reported for highly active OER materials,^[^ ^32^ ^]^ and the inclusion of the Walden inversion into the mechanistic breadth does not alter this picture.

I would like to recall that the volcano plot of Figure 1 is based on a data‐driven methodology without the need of explicit DFT calculations. Therefore, these activity volcano plots can be seen as a general guide to materials that may follow the mononuclear or the mononuclear‐Walden pathways. In the case of data‐driven approaches, it is important to benchmark the robustness of the observed results by a dedicated sensitivity analysis of the assumptions within the applied basis set.^[^ ^47^, ^48^ ^]^ Key parameters are the scaling relations between the ^*^OH and ^*^O as well as the ^*^OH and ^*^OOH adsorbates. The scaling‐relation intercept (SRI) between the ^*^OH and ^*^OOH intermediates, Δ*G*_2_ + Δ*G*_3_ = SRI, is well accepted to be on the order of (3.20 ± 0.20) eV^[^ ^22^ ^]^; though, smaller SRI values than the conventional value of 3.20 eV have been reported in the literature, which may refer to the consideration or neglection of the aqueous solvent in the DFT calculations.^[^ ^49^, ^50^, ^51^ ^]^ Therefore, the same mechanistic evaluation is conducted for SRI values of 3.00 and 2.80 eV, as illustrated in Figures S5 and S6 in Section S5 (Supporting Information), respectively. While the relative shape of the volcano curves are slightly different compared to SRI = 3.20 eV, the preference for a certain mechanistic pathway in dependence of the descriptor Δ*G*_1_ remains unchanged for SRI = 3.00 and 2.80 eV. In summary, the general picture of Figure 1 does not change if the ^*^OH versus ^*^OOH scaling relation is altered to a reasonable extent even if small changes in terms of the preferred mechanistic pathway can be observed for lower SRI values (cf. Figures S5 and S6, Supporting Information).

On the other hand, the scaling relation between the ^*^OH and ^*^O intermediates is less pronounced than the ^*^OH versus ^*^OOH scaling relation, implying that the ^*^OH versus ^*^O correlation might be prone to change the observed volcano curve. While Δ*G*_2_ = 2 × Δ*G*_1_ corresponds to the conventional assumption, in Figures S7 and S8 in Section S6 (Supporting Information), we inspect the impact of this scaling correlation on the volcano plot for Δ*G*_2_ = 2.3 × Δ*G*_1_ and Δ*G*_2_ = 1.5 × Δ*G*_1_, respectively. Similar to the discussion of a different SRI in the above, only minor changes relating to the binding‐energy regimes of Δ*G*_1_ for the preferred mechanism are observed. Yet, the analysis reveals that on a qualitative scale, the obtained results remain virtually constant in that the mononuclear‐Walden mechanism governs the volcano legs independent of applied electrode potential, the mononuclear pathway controls the volcano apex for small overpotentials, and both the mononuclear and mononuclear‐Walden mechanisms are observed for typical OER conditions at the volcano top. These findings illustrate that the chosen basis set relating to the scaling relations between the ^*^OH and ^*^O as well as the ^*^OH and ^*^OOH adsorbates is robust, but equally that the obtained results are of relevance to the entire OER material space. This finding underpins the importance of Walden‐inversion steps to the description of proton‐coupled electron transfer steps at electrified solid/ liquid interfaces.

Given that the robustness of the reported data‐driven methodology has been carefully counterchecked, in the next step, the concept of the Walden inversion is applied to other mechanistic pathways in the OER. Since our interest is mainly dedicated to the identification of highly active OER materials, we focus only on these mechanisms that appear at the volcano top. When inspecting the OER volcano plot in the approximation of *G*_max_(*U*) from a previous work (cf. Figures S2 and S3, Supporting Information),^[^ ^32^ ^]^ it turns out that a bifunctional description governs the volcano apex for small overpotentials, that is, *U* ≤ 1.40 V versus RHE. On the other hand, an oxide path comprising the formation of O_2_ by the chemical recombination of two adjacent ^*^OO ^*^OO groups is observed for *U* ≥ 1.60 V versus RHE at the volcano top (cf. Section S1 (Supporting Information)). Since the chemical recombination step already involves the presence of two adjacent active sites, the occurrence of a Walden inversion step in this pathway appears less likely due to steric reasons. Therefore, we do not focus on the oxide mechanism in this contribution, but rather address the bifunctional description, a mechanistic pathway requiring a proton acceptor site, ^*^O_A_, next to the catalytically active center as indicated by Equations (11)–(14):

(11) $$M+*O_{A}+H_{2}O\;\;\;\to M-\mathrm{OH}+*O_{A}+H^{+}+e^{-}\;\;\Delta G_{9}$$

(12) $$M-\mathrm{OH}+*O_{A}\;\;\;\to M-O+*O_{A}+H^{+}+e^{-}\;\;\;\;\Delta G_{10}$$

(13) $$M-O+*O_{A}+H_{2}O\;\to M-\mathrm{OO}+*{\mathrm{OH}}_{A}+H^{+}+e^{-}\;\Delta G_{11}$$

(14) $$M-\mathrm{OO}+*{\mathrm{OH}}_{A}\;\;\;\to M+*O_{A}+O_{2(g)}+H^{+}+e^{-}\;\;\;\Delta G_{12}$$

The surface oxygen atom next to the catalytically active site, ^*^O_A_, is involved in the splitting of the second water molecule on the oxygen‐covered surface (cf. Equation (13)) in that the ^*^OO rather the ^*^OOH adsorbate is formed since a proton is transferred to the neighboring acceptor site.

Similar to the mononuclear description, the bifunctional pathway can be rewritten by considering a Walden inversion step. Also in this case, the reference structure in the mechanism has to be changed to M‐OH. Due to the simultaneous evolution of gaseous oxygen and the adsorption of water in the form of an ^*^OH adsorbate, the unoccupied active site, M, is not observed in the pathway anymore. Equations (15)–(18) summarize the bifunctional‐Walden description:

(15) $$M-\mathrm{OH}\;+*O_{A}\;\;\;\to M-O+*O_{A}+H^{+}+e^{-}\;\;\;\;\Delta G_{13}$$

(16) $$M-O+*O_{A}+H_{2}O\;\;\to M-\mathrm{OO}+*{\mathrm{OH}}_{A}+H^{+}+e^{-}\;\;\;\Delta G_{14}$$

(17) $$M-\mathrm{OO}+*{\mathrm{OH}}_{A}\;\;\;\to M-\mathrm{OO}+*O_{A}+H^{+}+e^{-}\;\;\;\Delta G_{15}$$

(18) $$M-\mathrm{OO}+*O_{A}+H_{2}O\;\to \mathrm{HO}-M+*O_{A}+O_{2(g)}+H^{+}+e^{-}\;\Delta G_{16}$$

In this case, Equation (18) refers to the Walden inversion step comprising the concurrent desorption of the product and adsorption of the reactant. Like the comparison of the mononuclear and mononuclear‐Walden mechanisms, the bifunctional and bifunctional‐Walden pathways have two steps in common whereas the last two elementary steps in Equations (17) and (18) differ from the traditional description.

In Section S7 (Supporting Information), a rigorous thermodynamic analysis for the bifunctional and the bifunctional‐Walden mechanisms is conducted to derive the activity measure *G*_max_(*U*) in a potential‐dependent fashion. This allows compiling an activity volcano plot in Figure S9 (Supporting Information) to understand the mechanistic trends of these competing pathways. Contrary to the comparison of the mononuclear and mononuclear‐Walden mechanisms (cf. Figure 1), for low overpotentials the bifunctional and bifunctional‐Walden pathways are favored at the right volcano leg and left volcano leg including apex, respectively. Even if both mechanisms compete with increasing overpotential on the right‐hand side of the volcano, the apex of the OER volcano is governed by the bifunctional‐Walden description rather than the bifunctional mechanism (cf. Figure S9, Supporting Information).

**Figure 2:**
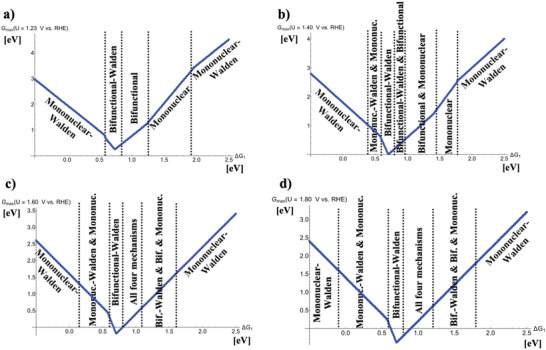
Potential‐dependent volcano plots for the mononuclear, mononuclear‐Walden, bifunctional, and bifunctional‐Walden pathways of the oxygen evolution reaction at a) *U* = 1.23 V versus RHE, b) *U* = 1.40 V versus RHE, c) *U* = 1.60 V versus RHE, and d) *U* = 1.80 V versus RHE. The energetically favored mechanisms in the approximation of *G*_max_(*U*) as a potential‐dependent activity descriptor are indicated in dependence of the adsorption free energy of the ^*^OH intermediate, Δ*G*_1_. To derive the volcano curves, the following scaling relations are considered: Δ*G*_2_ + Δ*G*_3_ = 3.20 eV and Δ*G*_2_ = 2 × Δ*G*_1_.

While the previous activity volcano plots of Figure 1 and Figure S9 (Supporting Information) have aimed to comprehend the impact of the Walden inversion on the OER volcano curve for a single mechanism, a real‐world electrocatalyst may not necessarily follow a single mechanistic description.^[^ ^52^, ^53^, ^54^, ^55^, ^56^ ^]^ Therefore, we combine the mononuclear and bifunctional pathways including the Walden inversion steps into an activity volcano plot, depicted in **Figure** **2**.

Figure 2 illustrates the mechanistic complexity of the OER if several mechanistic pathways are considered in the volcano. While for equilibrium conditions, *U* = 1.23 V versus RHE, each mechanism governs the activity volcano in a specific free‐energy regime, with increasing electrode potential several mechanisms compete, and a variety of different sections in the volcano are visible. Particularly for typical OER conditions, that is, *U* = 1.60 V versus RHE, all four mechanisms are in competition at the right‐hand side of the volcano close to the apex, and the bifunctional‐Walden mechanism is preferred at the volcano apex. Mechanistic pathways consisting of Walden steps have not been used for the modeling of OER catalysts in DFT studies so far, but their importance to the theoretical description of highly active materials is evident based on the presented generalized activity volcano plots. As a fundamental consequence, most theoretical studies aiming at the identification of high‐performance electrocatalysts may be erroneous since the reaction mechanism, which may be occurring under operational conditions, has not been considered in the underlying model. This finding underpins the need of dedicated mechanistic studies beyond the common assumption of traditional mechanisms.^[^ ^57^ ^]^

Finally, I would like to pinpoint a few subtleties of the present study and its implication to electrocatalysis. First, the present discussion of OER activity volcano plots relies on a data‐driven strategy, and thus, does not contain explicit DFT calculations. As such, it is not the aim of this study to predict a material that follows the bifunctional‐Walden mechanism, but rather I would like to convey to the community that more detailed mechanistic studies are needed for the OER, and I am convinced that this statement can be generalized to any electrocatalytic process. The main limitation of theoretical studies in the realm of materials development or the identification of limiting steps by volcano plots, besides other aspects such as the treatment of solvation, the choice of canonical or grand canonical approaches to obtain adsorption‐free energies, or the application of suitable activity descriptors,^[^ ^58^, ^59^, ^60^, ^61^, ^62^ ^]^ refers to the assumption of the mechanistic pathways, and the electrocatalytic activity can only be discussed based on the mechanisms considered in the underlying model. The inclusion of Walden inversion steps into mechanistic investigations of proton‐coupled electron transfer steps at electrified solid/ liquid interfaces may advance the theoretical discipline of electrocatalysis to obtain a more comprehensive picture of the elementary reaction steps during catalytic operation.

On the other hand, it should be noted that the occurrence of Walden inversion steps relies on the precondition that ^*^OH groups are available under OER conditions. One opportunity to countercheck this aspect is to inspect whether the formation of the ^*^OH adsorbate refers to the PDS. Given that applied electrode potentials of *U* > 1.50 V versus RHE are required to obtain a reasonable OER current density, these harsh anodic conditions should ultimately lead to an oxidation of metal surface atoms to form ^*^OH or ^*^O surface groups. Following the work by Calle‐Vallejo and coworkers,^[^ ^59^ ^]^ the formation of the ^*^OH adsorbate constitutes the PDS in <12% by referring to a large data set of materials ranging from transition‐metal oxides, metal oxides, perovskites, porphyrins, and functionalized graphitic materials. Electrocatalysts having the formation of the ^*^OH adsorbate as PDS are located at the volcano legs rather than at the volcano apex so that the general conclusions of this work relating to the reported Walden inversion steps for highly active materials are not affected. Though, it must be noted that the reported Walden mechanisms at the volcano legs for inactive catalysts (cf. Figure 2) only hold true if materials in this binding‐energy regime are not limited by the formation of ^*^OH surface groups. This is the case if the free‐energy change Δ*G*_1_ (cf. Equation (1)) exceeds the applied electrode potential when translated to a potential scale (right leg of the volcano). Despite this shortcoming, the focus of the present work is on highly active catalysts and their correct mechanistic description, and the above preconditions do not alter the importance of Walden‐type mechanisms at the volcano apex or the left volcano leg. It should also be noted that the formation of the ^*^OH adsorbate is not reconciled with the RDS under typical OER conditions,^[^ ^63^ ^]^ and thus, the formation of ^*^OH groups is never kinetically limiting. Therefore, it can be concluded that the precondition of ^*^OH groups on the catalyst surface is even fulfilled if the formation of ^*^OH groups is reconciled with the PDS. For a more detailed discussion on this matter, we refer to Section S8 (Supporting Information).

The observed importance of Walden inversion steps relating to the OER activity volcano spans a bridge to stability investigations of electrocatalysts. Let me emphasize that the present descriptor‐based volcano study in the approximation of *G*_max_(*U*) focuses on the electrocatalytic activity whereas, hitherto, catalyst decomposition or structural reorganizations cannot be tackled by our data‐driven models due to the lack of scaling relations and mechanistic knowledge for catalyst decomposition. The notion of the Walden inversion as a motif for product formation in the OER has been motivated based on a recent work on catalyst stability, recalling that Hess and Over reported that a Walden inversion step plays a key role in the decomposition of a RuO_2_ electrode.^[^ ^40^ ^]^ While the present study cannot comment on the frequently stated hypothesis that higher electrocatalytic activity is correlated with a lower stability of the active center, yet it can be concluded unambiguously based on the volcano plots of Figures 1 and 2 in conjunction with the study by Hess and Over that the Walden inversion is of fundamental importance to comprehend the electrocatalytic activity and stability of electrocatalysts both. Future studies should therefore incorporate Walden inversion steps simultaneously into the modeling of product‐forming (activity) and degradation (stability) pathways to unravel their relevance to electrocatalytic processes at electrified solid/ liquid interfaces since this may broaden our knowledge of electrocatalysts under dynamic reaction conditions.

## 3 Conclusion

Oxygen evolution (OER) is also denoted as the enigma in water electrolysis since neither profound mechanistic knowledge nor an ideal catalyst in terms of activity and stability has been revealed so far.^[^ ^64^ ^]^ While theoretical considerations in the density functional theory approximation in conjunction with descriptor‐based analyses in the realm of volcano plots fuel hope to unravel mechanistic feature as well as to steer catalyst design, hitherto, no major breakthrough has been reported. One of the reasons for this finding may be the fact that most computational studies rely on simplified mechanistic models in that only the energetics of a single reaction mechanisms is considered to approximate the electrocatalytic activity by the thermodynamic analysis of adsorption‐free energies. While the importance of various mechanistic pathways and a switch in the energetically favored mechanism with increasing overpotential has been reported for highly active OER materials only recently,^[^ ^32^ ^]^ the present work sheds light on the significance on Walden inversion steps to the mechanistic description of the OER.

Stimulated by a recent work on the relevance of the Walden inversion to the decomposition of a RuO_2_ electrode,^[^ ^40^ ^]^ activity volcano plots for the OER in dependence of various mechanistic pathways with the inclusion of Walden inversion steps are derived by assessing the electrocatalytic activity by the descriptor *G*_max_(*U*), a potential‐dependent activity measure based on the free‐energy span model.^[^ ^31^ ^]^ This type of analysis is achieved by a data‐driven methodology in that the energetics of the considered mechanistic pathways is related by scaling relations to a basis set of adsorption‐free energies.^[^ ^45^ ^]^ The as‐derived activity volcano plots for the OER reveal that the Walden inversion, albeit so far not considered for the modeling of OER pathways, plays an important role in the anodic formation of gaseous oxygen since the entire activity volcano is governed by Walden inversion steps under typical reaction conditions of *U* = 1.60 V versus RHE. This finding calls for a change in the mindset in that future computational studies aiming at the modeling of proton‐coupled electron transfer steps at electrified solid/ liquid interfaces need to incorporate the opportunity of Walden inversion steps into their mechanistic investigations to gain an atomic‐scale understanding of the elementary processes that govern the electrocatalytic activity and stability.

## Conflict of Interest

The authors declare no conflict of interest.

## Supporting information

Supporting Information

## Data Availability

The data that support the findings of this study are available from the corresponding author upon reasonable request.
