# Supplementary material for: Importance of the Walden Inversion for the Activity Volcano Plot of Oxygen Evolution
Source: Adv Sci (Weinh). 2023 Oct 30;10(36):2305505. doi: 10.1002/advs.202305505 (PMC10754130; doi:10.1002/advs.202305505)
Supplement: Supplementary file 1 — Supporting Information [file ADVS-10-2305505-s001.pdf]

ADVANCED SCIENCE
Open Access

Supporting Information

for *Adv. Sci.*, DOI 10.1002/advs.202305505

Importance of the Walden Inversion for the Activity Volcano Plot of Oxygen Evolution

*Kai S. Exner**

Supporting Information

Importance of the Walden inversion for the activity volcano plot of oxygen evolution

Kai S. Exner[1,2,3,*]

[1] *University Duisburg-Essen, Faculty of Chemistry, Theoretical Inorganic Chemistry, Universitätsstraße 5, 45141 Essen, Germany*

[2] *Cluster of Excellence RESOLV, Bochum, Germany*

[3] *Center for Nanointegration (CENIDE) Duisburg-Essen, Duisburg, Germany*

* Corresponding author: kai.exner@uni-due.de ORCID: 0000-0003-2934-6075

Keywords

OER; Walden inversion; reaction mechanism; volcano plot; descriptor approach

1 Volcano plots for oxygen evolution

The mononuclear description of the oxygen evolution reaction (OER), $2\ H_2O \rightarrow O_2 + 4\ H^+ + 4\ e^-$ with $U^0_{OER} = 1.23$ V *vs*. RHE (reversible hydrogen electrode), reads (cf. equation (1) – (4)) **[1]**:

$$M + H_2O \rightarrow M\text{-}OH + H^+ + e^- \qquad \Delta G_1 \qquad (1)$$

$$M\text{-}OH \rightarrow M\text{-}O + H^+ + e^- \qquad \Delta G_2 \qquad (2)$$

$$M\text{-}O + H_2O \rightarrow M\text{-}OOH + H^+ + e^- \qquad \Delta G_3 \qquad (3)$$

$$M\text{-}OOH \rightarrow M + O_{2(g)} + H^+ + e^- \qquad \Delta G_4 \qquad (4)$$

In equations (1) – (4), M denotes the catalytically active surface site (e. g., an undercoordinated metal atom), and the four OER free-energy changes meet the criterion of equation (5):

$$\Delta G_1 + \Delta G_2 + \Delta G_3 + \Delta G_4 = +4.92 \text{ eV } @U = 0 \text{ V } vs.\text{ RHE} \qquad (5)$$

The thermodynamic overpotential, η_{TD}, serving as the activity descriptor is given by equation (6):

$$\eta_{TD} = \max\{\Delta G_1 - 1.23 \text{ eV}; \Delta G_2 - 1.23 \text{ eV}; \Delta G_3 - 1.23 \text{ eV}; \Delta G_4 - 1.23 \text{ eV}\} / e \qquad (6)$$

Using the concept of scaling relation **[2]**, an activity volcano plot can be derived that depicts the electrocatalytic activity in the approximation of η_{TD} as a function of the descriptor ΔG_1 (cf. **Figure S1**). The elementary step with the largest free-energy change, corresponding to the activity measure η_{TD}, is also denoted as the potential-determining step (PDS). **Figure S1** reveals that either *OOH formation or *O formation governs the electrocatalytic activity at the left and right volcano legs, respectively, thereby making use of the tacit assumption that the PDS is equal to the rate-determining step **[3]**. Hence, the common approach to enhance the electrocatalytic

activity of electrode materials refers to the stabilization of the *OOH or *O adsorbates to lower the value of η_{TD}, depending on the position of the electrocatalyst in the volcano plot **[4]**.

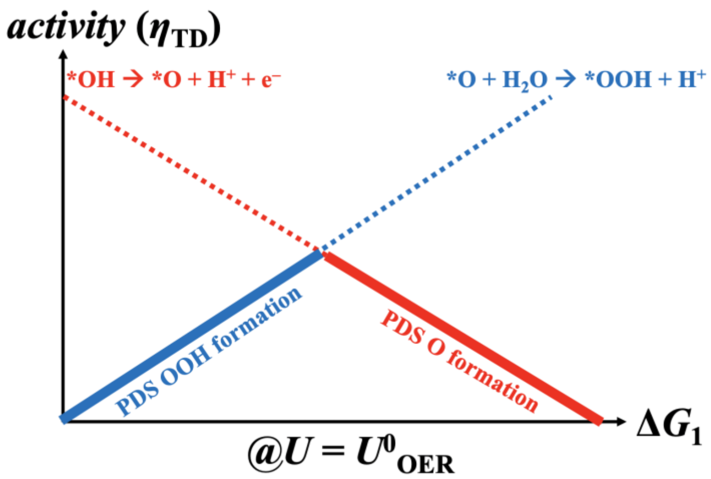

Figure S1. Generalized volcano plot for the oxygen evolution reaction based on the mononuclear mechanism under equilibrium conditions (U = 1.23 V *vs*. RHE). *OOH and *O formations are reconciled with the potential-dependent steps (PDS) at the left and right volcano legs, respectively. Figure reproduced with permission from reference **[5]**.

In a recent contribution, Exner reinvestigated the volcano plot of the OER **[5]**. In this context, not only the mononuclear mechanism but rather six different mechanistic descriptions were included into the model **[6-9]**. These pathways are listed in the following.

a) Mononuclear mechanism (cf. equations (1) – (4))

b) Bifunctional mechanism I (cf. equations (7) – (10))

$M + {*O_A} + H_2O \rightarrow M\text{-}OH + {*O_A} + H^+ + e^-$ ΔG_a (7)

$M\text{-}OH + {*O_A} \rightarrow M\text{-}O + {*O_A} + H^+ + e^-$ ΔG_b (8)

$M\text{-}O + {*O_A} + H_2O \rightarrow M\text{-}OO + {*OH_A} + H^+ + e^-$ ΔG_c (9)

$M\text{-}OO + {*OH_A} \rightarrow M + {*O_A} + O_{2(g)} + H^+ + e^-$ ΔG_d (10)

c) Bifunctional mechanism II (cf. equations (11) – (15))

$M + {*O_A} + H_2O \rightarrow M\text{-}OH + {*O_A} + H^+ + e^-$ ΔG_e (11)

$M\text{-}OH + {*O_A} \rightarrow M\text{-}O + {*O_A} + H^+ + e^-$ ΔG_f (12)

$M\text{-}O + {*O_A} + H_2O \rightarrow M\text{-}OOH + {*OH_A}$ ΔG_g (13)

${*OH_A} \rightarrow {*O_A} + H^+ + e^-$ ΔG_h (14)

$M\text{-}OOH \rightarrow M + O_{2(g)} + H^+ + e^-$ ΔG_i (15)

d) Binuclear mechanism (cf. equations (16) – (20))

$M + M + H_2O \rightarrow M\text{-}OH + M + H^+ + e^-$ ΔG_j (16)

$M\text{-}OH + M + H_2O \rightarrow M\text{-}OH + M\text{-}OH + H^+ + e^-$ ΔG_k (17)

$M\text{-}OH + M\text{-}OH \rightarrow M\text{-}O + M\text{-}OH + H^+ + e^-$ ΔG_l (18)

M-O + M-OH	$\rightarrow$ M-O + M-O + H^+ + e^-	ΔG_m	(19)
M-O + M-O	$\rightarrow$ M + M + $O_{2(g)}$	ΔG_n	(20)

e) Oxide mechanism (also denoted as *OO··*OO recombination mechanism, cf. equations (21) – (25))

M-O M-O + H_2O	$\rightarrow$ M-OOH M-O + H^+ + e^-	ΔG_o	(21)
M-OOH M-O + H_2O	$\rightarrow$ M-OOH M-OOH + H^+ + e^-	ΔG_p	(22)
M-OOH M-OOH	$\rightarrow$ M-OOH M-OO + H^+ + e^-	ΔG_q	(23)
M-OOH M-OO	$\rightarrow$ M-OO M-OO + H^+ + e^-	ΔG_r	(24)
M-OO M-OO	$\rightarrow$ M-O M-O + $O_{2(g)}$	ΔG_s	(25)

f) Two-electron water oxidation (cf. equations (26) – (28))

2 e^- water oxidation: 2 H_2O	$\rightarrow$ H_2O_2 + 2 H^+ + 2 e^-	U^0 = 1.76 V *vs*. RHE	(26)
M + H_2O	$\rightarrow$ M-OH + H^+ + e^-	ΔG_t	(27)
M-OH + H_2O	$\rightarrow$ M + H_2O_2 + H^+ + e^-	ΔG_u	(28)

The free-energy changes ΔG_α (α = a, …, u) of the various mechanistic pathways are related by a rigorous thermodynamic treatment **[10]** to the free energies of the reaction intermediates, thereby making use of the scaling relations between the *OH and *O as well as the *OH and *OOH adsorbates **[2]**. Knowledge of the reaction intermediates' free energies enables determining the activity descriptor $G_{max}(U)$ **[11]**, an advanced activity measure based on the idea of the free-energy span model **[12]**. In contrast to η_{TD}, $G_{max}(U)$ offers a potential-dependent description of the energetics, and thus, enables approximation of the electrocatalytic activity not only under equilibrium conditions (U = 1.23 V *vs*. RHE) but equally under OER conditions encountered during experimental measurements. **Figures S2-S3** show potential-dependent volcano plots for the OER, thereby taking all the above pathways into consideration.

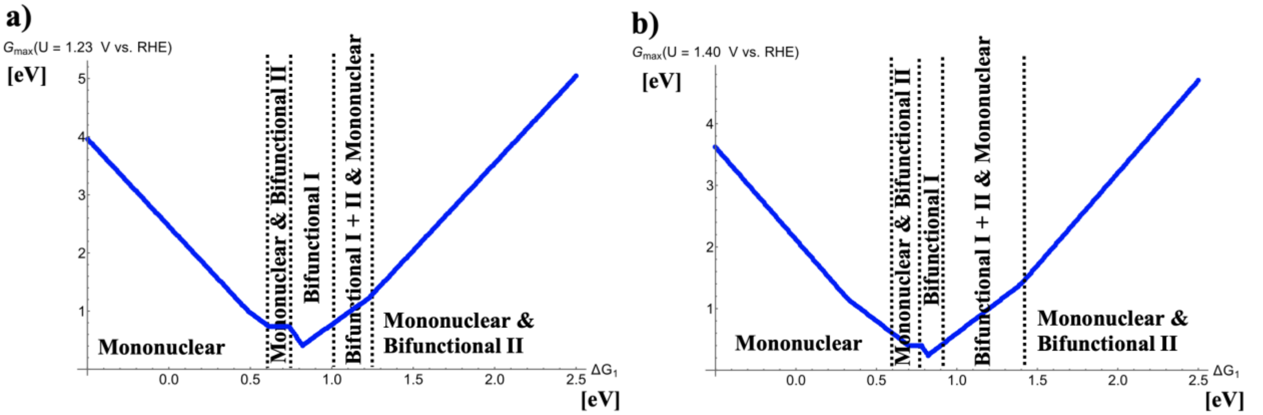

Figure S2. Potential-dependent volcano plots for various pathways of the oxygen evolution reaction at **a)** U = 1.23 V *vs*. RHE and **b)** U = 1.40 V *vs*. RHE. The energetically favored mechanisms in the approximation of $G_{max}(U)$ as a potential-dependent activity measure are indicated in dependence of the adsorption free energy of the *OH intermediate, ΔG_1. To derive the volcano curves, the following scaling relations are considered: $\Delta G_2 + \Delta G_3$ = 3.20 eV and $\Delta G_2 = 2 \times \Delta G_1$. Figure reproduced with permission from reference **[5]**.

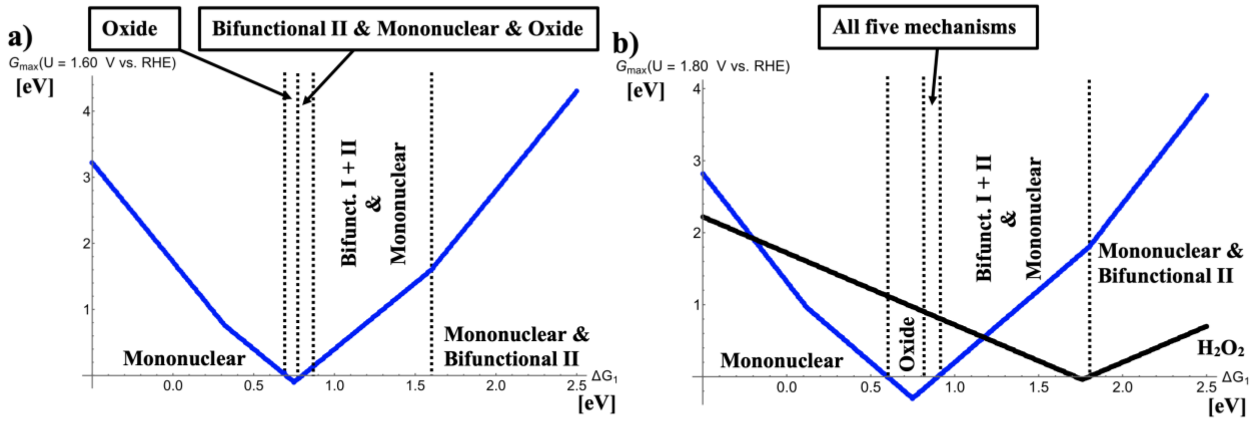

Figure S3. Potential-dependent volcano plots for various pathways of the oxygen evolution reaction at **a)** U = 1.60 V *vs*. RHE and **b)** U = 1.80 V *vs*. RHE. The energetically favored mechanisms in the approximation of $G_{max}(U)$ as a potential-dependent activity measure are indicated in dependence of the adsorption free energy of the *OH intermediate, ΔG_1. The black volcano curve in panel **b)** refers to the two-electron water oxidation with H_2O_2 as the main product. To derive the volcano curves, the following scaling relations are considered: $\Delta G_2 + \Delta G_3$ = 3.20 eV and $\Delta G_2 = 2 \times \Delta G_1$. Figure reproduced with permission from reference **[5]**.

Figures S2-S3 illustrate unambiguously that various mechanistic pathways govern the OER activity volcano. The mononuclear description is only a good approximation for the volcano legs, but not for the volcano apex where the highly active materials are situated. Most notably, it becomes evident that highly active OER electrocatalysts reveal a change in the reaction mechanism with increasing electrode potential, a situation that, hitherto, has been completely overlooked in the modeling of OER materials. For further discussion, the interested reader is referred to reference **[5]**.

It is noticeable that the volcano curves in **Figures S2-S3** reveal kinks at the volcano legs. This finding is related to the fact that the volcano slope does not only change at the volcano apex, but even is prone to alter at the volcano legs. A change in the volcano slope at the legs is observed if another mechanistic pathway is energetically preferred or if another elementary step becomes the limiting one. For a detailed discussion on this matter and its implication to electrocatalysis, the interested reader is referred to references **[13,14]**.

2 Thermodynamic analysis of the mononuclear mechanism

The presented modeling approach relies on an in-house methodology that connects the adsorption free energies of the intermediate species in the reaction mechanisms to the electrocatalytic activity by the descriptor $G_{max}(U)$ to compile volcano curves for oxygen evolution **[14]**. In the following, this procedure is illustrated on the example of the mononuclear mechanism (cf. equations (1) – (4)). The free energies of the reaction intermediates *, *OH, *O, and *OOH in dependence of the applied electrode potential are given by equations (29) – (33):

$$G_M(U) = 0 \quad (29)$$

$$G_{M\text{-}OH}(U) = \Delta G_1 - 1 \times e \times U \quad (30)$$
$$G_{M\text{-}O}(U) = \Delta G_1 + \Delta G_2 - 2 \times e \times U \quad (31)$$
$$G_{M\text{-}OOH}(U) = \Delta G_1 + \Delta G_2 + \Delta G_3 - 3 \times e \times U \quad (32)$$
$$G_{M+O2}(U) = + 4.92\ eV - 4 \times e \times U \quad (33)$$

By considering the scaling relations of equations (34) and (35),

$$\Delta G_2 + \Delta G_3 = SRI \quad (34)$$
$$\Delta G_2 = 2 \times \Delta G_1 \quad (35)$$

the energetics of the intermediate states are:

$$G_{M}(U) = 0 \quad (36)$$
$$G_{M\text{-}OH}(U) = \Delta G_1 - 1 \times e \times U \quad (37)$$
$$G_{M\text{-}O}(U) = 3 \times \Delta G_1 - 2 \times e \times U \quad (38)$$
$$G_{M\text{-}OOH}(U) = \Delta G_1 + SRI - 3 \times e \times U \quad (39)$$
$$G_{M+O2}(U) = + 4.92\ eV - 4 \times e \times U \quad (40)$$

For the scaling-relation intercept (SRI), we adopt a benchmark value of SRI = 3.20 eV and consider SRI = 3.00 eV and 2.80 eV in the realm of sensitivity analyses **[14]**. For the scaling relation between the *OH and *O adsorbates (cf. equation (35)), different correlations are considered for sensitivity analyses, namely $\Delta G_2 = 2.3 \times \Delta G_1$ and $\Delta G_2 = 1.5 \times \Delta G_1$. The free-energy change ΔG_1 serves as the descriptor on the x axis in the activity volcano, and it is varied within the free-energy regime of ΔG_1 = [–0.50, 2.50] eV with a step size of 0.01 eV. Based on the energetics of the intermediate states, the descriptor $G_{max}(U)$ is evaluated by considering all possible free-energy spans between the reaction intermediates:

$$G_{max}(U) = \max\{G_{M\text{-}OH}(U) - G_{M}(U); G_{M\text{-}O}(U) - G_{M}(U); G_{M\text{-}OOH}(U) - G_{M}(U); G_{M\text{-}O}(U) - G_{M\text{-}OH}(U); G_{M\text{-}OOH}(U) - G_{M\text{-}OH}(U); G_{M+O2}(U) - G_{M\text{-}OH}(U); G_{M\text{-}OOH}(U) - G_{M\text{-}O}(U); G_{M+O2}(U) - G_{M\text{-}O}(U); G_{M+O2}(U) - G_{M\text{-}OOH}(U)\} \quad (41)$$

Volcano curves arise by plotting $G_{max}(U)$ as a function of ΔG_1 at a constant applied electrode potential, U, see **Figures S2-S3** as a prototypical example for the entire breadth of OER mechanisms.

3 Thermodynamic analysis of the mononuclear-Walden mechanism

The mononuclear-Walden mechanism reads:

$$M\text{-}OH \rightarrow M\text{-}O + H^+ + e^- \quad \Delta G_5 \quad (42)$$
$$M\text{-}O + H_2O \rightarrow M\text{-}OOH + H^+ + e^- \quad \Delta G_6 \quad (43)$$
$$M\text{-}OOH \rightarrow M\text{-}OO + H^+ + e^- \quad \Delta G_7 \quad (44)$$
$$M\text{-}OO + H_2O \rightarrow M\text{-}OH + O_{2(g)} + H^+ + e^- \quad \Delta G_8 \quad (45)$$

The free energies of the reaction intermediates *OH, *O, *OOH, and *OO in dependence of the applied electrode potential are given by equations (29) – (33):

$$G_{M\text{-}OH}(U) = 0 \quad (46)$$
$$G_{M\text{-}O}(U) = \Delta G_5 \qquad - 1 \times e \times U \quad (47)$$
$$G_{M\text{-}OOH}(U) = \Delta G_5 + \Delta G_6 \qquad - 2 \times e \times U \quad (48)$$
$$G_{M\text{-}OO}(U) = \Delta G_5 + \Delta G_6 + \Delta G_7 \qquad - 3 \times e \times U \quad (49)$$
$$G_{M\text{-}OH+O2}(U) = \qquad + 4.92\text{ eV} \qquad - 4 \times e \times U \quad (50)$$

Due to $\Delta G_5 = \Delta G_2$ and $\Delta G_6 = \Delta G_3$, the energetics of the mononuclear-Walden mechanism can be related to the scaling relations of equations (34) and (35). Additionally, it can be shown that $\Delta G_7 = \Delta G_2$, and thus we obtain:

$$G_{M\text{-}OH}(U) = 0 \quad (51)$$
$$G_{M\text{-}O}(U) = 2 \times \Delta G_1 \qquad - 1 \times e \times U \quad (52)$$
$$G_{M\text{-}OOH}(U) = \qquad + \text{SRI} \qquad - 2 \times e \times U \quad (53)$$
$$G_{M\text{-}OO}(U) = 2 \times \Delta G_1 \qquad + \text{SRI} \qquad - 3 \times e \times U \quad (54)$$
$$G_{M\text{-}OH+O2}(U) = \qquad + 4.92\text{ eV} \qquad - 4 \times e \times U \quad (55)$$

Based on the intermediate states' energetics, the free-energy spans for the mononuclear-Walden mechanism can be defined to derive $G_{max}(U)$:

$$G_{max}(U) = \max\{G_{M\text{-}O}(U) - G_{M\text{-}OH}(U);\ G_{M\text{-}OOH}(U) - G_{M\text{-}OH}(U);\ G_{M\text{-}OO}(U) - G_{M\text{-}OH}(U);$$
$$G_{M\text{-}OOH}(U) - G_{M\text{-}O}(U);\ G_{M\text{-}OO}(U) - G_{M\text{-}O}(U);\ G_{M\text{-}OH+O2}(U) - G_{M\text{-}O}(U);$$
$$G_{M\text{-}OO}(U) - G_{M\text{-}OOH}(U);\ G_{M\text{-}OH+O2}(U) - G_{M\text{-}OOH}(U);\ G_{M\text{-}OH+O2}(U) - G_{M\text{-}OO}(U)\} \quad (56)$$

4 Volcano plot for the mononuclear and mononuclear-Walden mechanisms

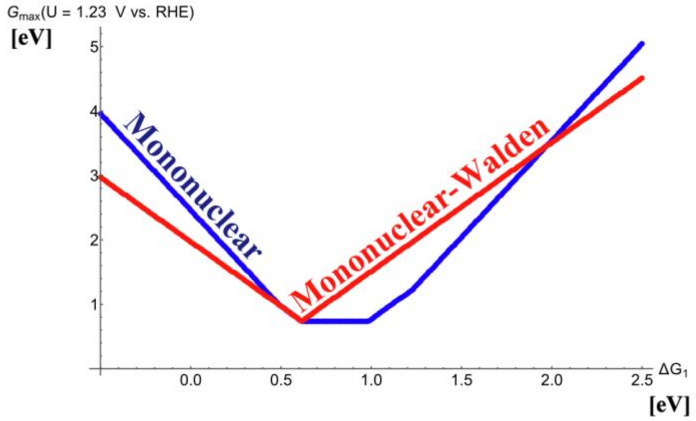

Figure S4. Potential-dependent volcano plot for the mononuclear (blue) and mononuclear-Walden (red) mechanisms of oxygen evolution at U = 1.23 V *vs.* RHE. To derive the volcano lines, the activity measure $G_{max}(U)$ (cf. equations (41) and (56)) is evaluated for the intermediate states of the mechanistic pathways (cf. equations (36) – (40) and (51) – (55)) in dependence of the descriptor ΔG_1, which is varied between –0.5 eV and +2.5 eV in steps of 0.01 eV. In the analysis, the following scaling relations are considered: $\Delta G_2 + \Delta G_3$ = 3.20 eV and $\Delta G_2 = 2 \times \Delta G_1$.

The energetics of the mononuclear and mononuclear-Walden mechanisms is compiled in a single activity volcano plot based on the above modeling approach to determine the energetically favored pathway in dependence of the descriptor ΔG_1. This comprises that, based on the obtained values of $G_{max}(U)$ for the mononuclear and mononuclear-Walden mechanisms (cf. equations (41) and (56), respectively), the minimum $G_{max}(U)$ value is plotted as a function of ΔG_1. **Figure S4** indicates the raw data for the OER volcano plot at U = 1.23 V *vs*. RHE, and its analysis culminates into **Figure 1a** of the main text where only the energetically favored pathway (minimum value of $G_{max}(U)$) is shown.

The following free-energy spans limit the OER volcano plot at U = 1.23 V *vs*. RHE (cf. **Figure 1a** of the main text):

a) – 0.5 eV < ΔG_1 < 0.62 eV (mononuclear-Walden mechanism): $G_{M\text{-}OOH}(U) - G_{M\text{-}O}(U)$
b) 0.62 eV < ΔG_1 < 0.99 eV (mononuclear mechanism): $G_{M\text{-}OOH}(U) - G_{M\text{-}OH}(U)$
c) 0.99 eV < ΔG_1 < 1.23 eV (mononuclear mechanism): $G_{M\text{-}O}(U) - G_{M\text{-}OH}(U)$
d) 1.23 eV < ΔG_1 < 1.97 eV (mononuclear mechanism): $G_{M\text{-}O}(U) - G_{M}(U)$
e) 1.97 eV < ΔG_1 < 2.50 eV (mononuclear-Walden mechanism): $G_{M\text{-}OO}(U) - G_{M\text{-}OH}(U)$

Changes in the preferred mechanisms and limiting steps cause the observation of kinks at the legs of the OER volcano curve, as outlined in section 1 of the supplemental.

5 Sensitivity analysis of the *OH *vs*. *OOH scaling relation

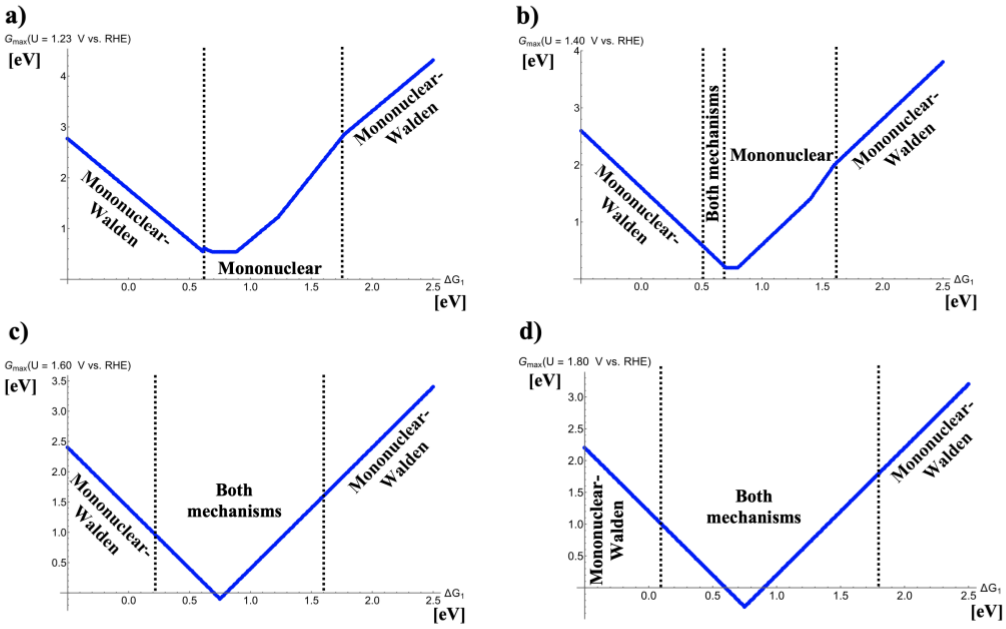

Figure S5. Potential-dependent volcano plots for the mononuclear and the mononuclear-Walden pathways of the oxygen evolution reaction at **a)** U = 1.23 V *vs*. RHE, **b)** U = 1.40 V *vs*. RHE, **c)** U = 1.60 V *vs*. RHE, and **d)** U = 1.80 V *vs*. RHE. The energetically favored mechanisms in the approximation of $G_{max}(U)$ as a potential-dependent activity measure are indicated in dependence of the adsorption free energy of the *OH intermediate, ΔG_1. To derive the volcano curves, the following scaling relations are considered: $\Delta G_2 + \Delta G_3$ = 3.00 eV and $\Delta G_2 = 2 \times \Delta G_1$.

Given that smaller scaling-relation intercepts (SRI) than the conventional value of 3.20 eV have been reported in the literature **[15-17]**, activity volcano plots for the mononuclear and mononuclear-Walden mechanisms adopting SRI = 3.00 eV and 2.80 eV are derived. The results are compiled in **Figures S5-S6**.

Compared to **Figure 1** of the main text (SRI = 3.20 eV), there are only minor alterations in terms of the preferred OER mechanism visible. For instance, for SRI = 2.80 eV, the mononuclear and mononuclear-Walden mechanisms compete already at U = 1.40 V *vs*. RHE at the volcano apex whereas for SRI = 3.20 eV and SRI = 3.00 eV, mechanistic competition at the volcano top is observed at U = 1.60 V *vs*. RHE. Overall, the general mechanistic trends are not changed when the SRI is reduced to smaller values. Notably, the volcano legs are always described by the same mechanism, namely the mononuclear-Walden description, and the volcano apex reveals competition between the two pathways under typical OER conditions of U = 1.60 V *vs*. RHE. This finding underpins the robustness of the presented analysis despite the assumption of scaling relations in the evaluation of adsorption free energies.

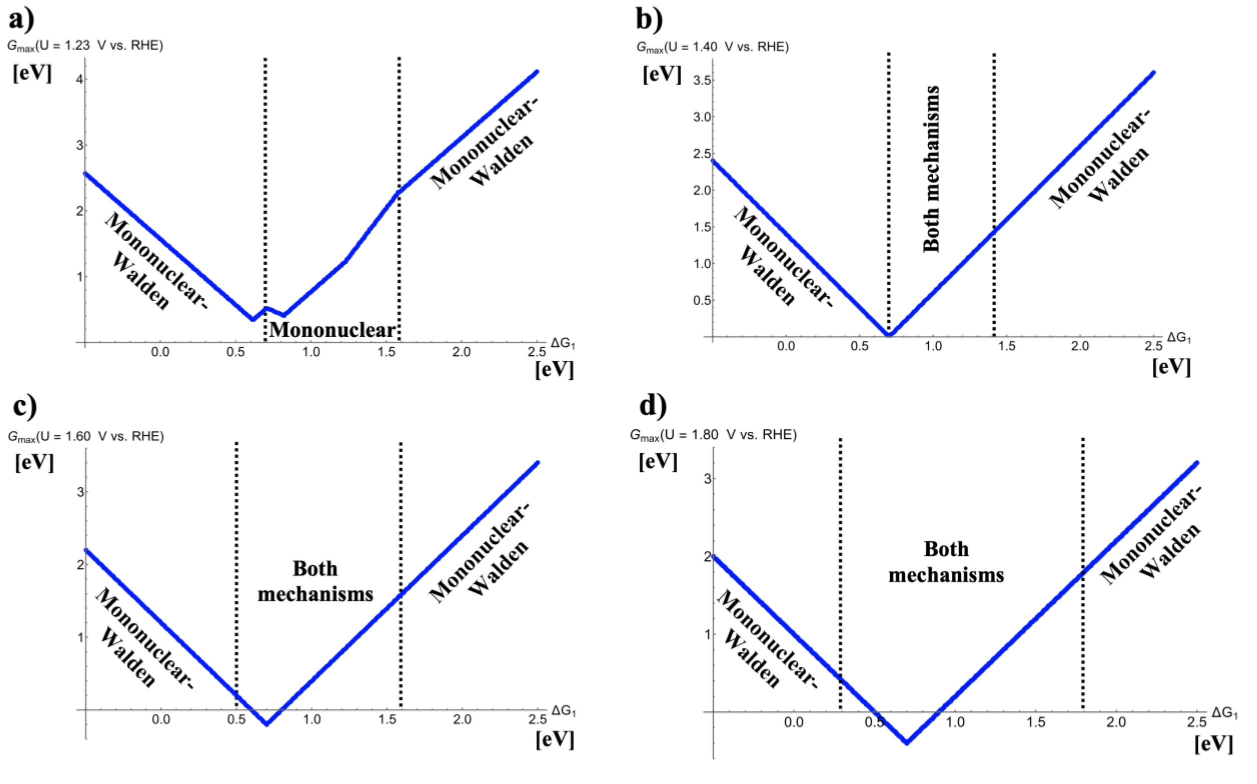

Figure S6. Potential-dependent volcano plots for the mononuclear and the mononuclear-Walden pathways of the oxygen evolution reaction at **a)** U = 1.23 V *vs*. RHE, **b)** U = 1.40 V *vs*. RHE, **c)** U = 1.60 V *vs*. RHE, and **d)** U = 1.80 V *vs*. RHE. The energetically favored mechanisms in the approximation of $G_{max}(U)$ as a potential-dependent activity measure are indicated in dependence of the adsorption free energy of the *OH intermediate, ΔG_1. To derive the volcano curves, the following scaling relations are considered: $\Delta G_2 + \Delta G_3$ = 2.80 eV and $\Delta G_2 = 2 \times \Delta G_1$.

6 Sensitivity analysis of the *OH *vs*. *O scaling relation

While the scaling relation between the *OH and *OOH intermediates is quite robust (cf. equation (34)), the opposite case is encountered with the scaling relation between the *OH and *O adsorbates (cf. equation (35)). This is the main reason why a sensitivity analysis is executed to demonstrate that the observed OER volcano trends hold true even if the energetics of the *OH and *O intermediates is altered to a reasonable extent. While **Figure S7** compiles an activity volcano for an enlarged slope ($\Delta G_2 = 2.3 \times \Delta G_1$), **Figures S8** indicate the opposite scenario of a reduced slope ($\Delta G_2 = 1.5 \times \Delta G_1$) between the free energies ΔG_2 and ΔG_1, adopting the mononuclear and mononuclear-Walden mechanisms as a case study.

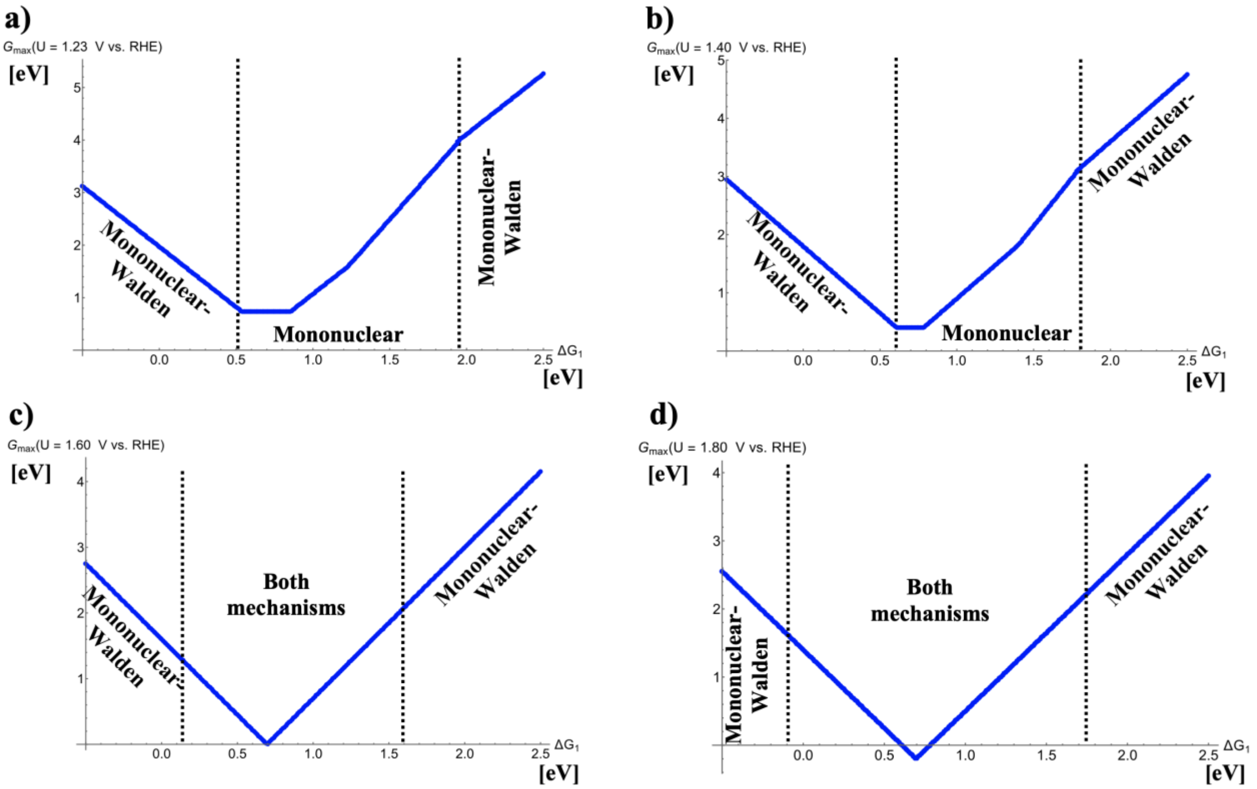

Figure S7. Potential-dependent volcano plots for the mononuclear and the mononuclear-Walden pathways of the oxygen evolution reaction at **a)** U = 1.23 V *vs*. RHE, **b)** U = 1.40 V *vs*. RHE, **c)** U = 1.60 V *vs*. RHE, and **d)** U = 1.80 V *vs*. RHE. The energetically favored mechanisms in the approximation of $G_{\text{max}}(U)$ as a potential-dependent activity measure are indicated in dependence of the adsorption free energy of the *OH intermediate, ΔG_1. To derive the volcano curves, the following scaling relations are considered: $\Delta G_2 + \Delta G_3 = 3.20$ eV and $\Delta G_2 = 2.3 \times \Delta G_1$.

Figures S7-S8 confirm that the volcano legs of the OER volcano are governed by the mononuclear-Walden description. This finding coincides with the volcano plot of the main text (cf. **Figure 1**). At the volcano apex, the mononuclear mechanism is preferred for small overpotentials, $U \leq 1.40$ V *vs*. RHE, whereas for large overpotentials, $U \geq 1.60$ V *vs*. RHE, the

two mechanistic descriptions compete. For SRI = 2.80 eV, a competition between the two pathways is already visible at smaller applied electrode potentials (cf. **Figures S8**), yet competition at the volcano top becomes visible only at U = 1.60 V *vs*. RHE, in agreement with SRI = 3.00 and 3.20 eV. In summary, the sensitivity analysis reveals that the obtained results relating to the OER volcano curve of the mononuclear and mononuclear-Walden descriptions is robust despite the assumption of the scaling relation between the *OH and *O intermediates.

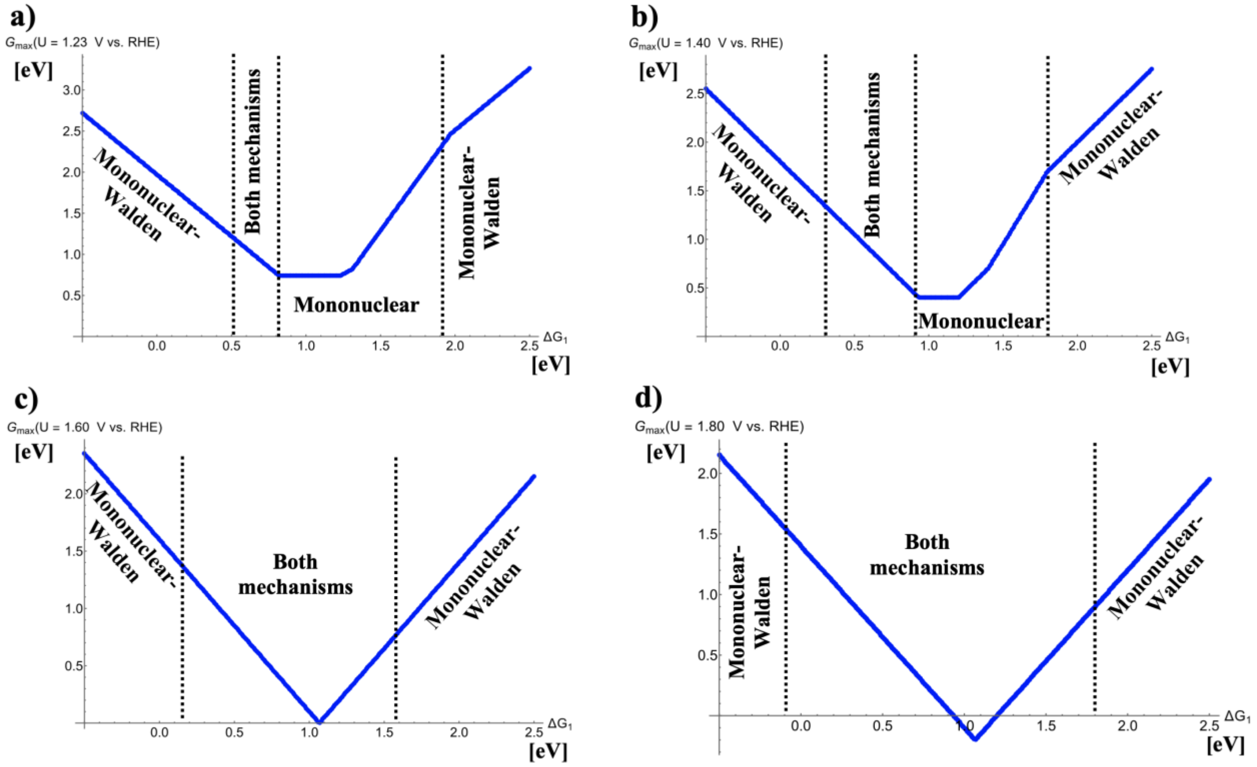

Figure S8. Potential-dependent volcano plots for the mononuclear and the mononuclear-Walden pathways of the oxygen evolution reaction at **a)** U = 1.23 V *vs*. RHE, **b)** U = 1.40 V *vs*. RHE, **c)** U = 1.60 V *vs*. RHE, and **d)** U = 1.80 V *vs*. RHE. The energetically favored mechanisms in the approximation of $G_{max}(U)$ as a potential-dependent activity measure are indicated in dependence of the adsorption free energy of the *OH intermediate, ΔG_1. To derive the volcano curves, the following scaling relations are considered: $\Delta G_2 + \Delta G_3$ = 3.20 eV and $\Delta G_2 = 1.5 \times \Delta G_1$.

7 Thermodynamic analysis of the bifunctional and bifunctional-Walden mechanisms

The bifunctional mechanism reads:

$$\text{M} + {}^*\text{O}_\text{A} + \text{H}_2\text{O} \rightarrow \text{M-OH} + {}^*\text{O}_\text{A} + \text{H}^+ + \text{e}^- \qquad \Delta G_9 \qquad (57)$$

$$\text{M-OH} + {}^*\text{O}_\text{A} \rightarrow \text{M-O} + {}^*\text{O}_\text{A} + \text{H}^+ + \text{e}^- \qquad \Delta G_{10} \qquad (58)$$

$$\text{M-O} + {}^*\text{O}_\text{A} + \text{H}_2\text{O} \rightarrow \text{M-OO} + {}^*\text{OH}_\text{A} + \text{H}^+ + \text{e}^- \qquad \Delta G_{11} \qquad (59)$$

$$\text{M-OO} + {}^*\text{OH}_\text{A} \rightarrow \text{M} + {}^*\text{O}_\text{A} + \text{O}_{2(g)} + \text{H}^+ + \text{e}^- \qquad \Delta G_{12} \qquad (60)$$

The free energies of the reaction intermediates *, *OH, *O, and *OO in dependence of the applied electrode potential are given by equations (61) – (65):

$$G_{M}(U) = 0 \tag{61}$$
$$G_{M\text{-}OH}(U) = \Delta G_9 - 1 \times e \times U \tag{62}$$
$$G_{M\text{-}O}(U) = \Delta G_9 + \Delta G_{10} - 2 \times e \times U \tag{63}$$
$$G_{M\text{-}OO}(U) = \Delta G_9 + \Delta G_{10} + \Delta G_{11} - 3 \times e \times U \tag{64}$$
$$G_{M+O2}(U) = + 4.92\ \text{eV} - 4 \times e \times U \tag{65}$$

Due to $\Delta G_9 = \Delta G_1$ and $\Delta G_{10} = \Delta G_2$, the energetics of the mononuclear-Walden mechanism can be related to the scaling relations of equations (34) and (35). Please note that by adopting these correlations, we make use of the tacit assumption that the adsorption energetics is independent of the chemical environment. This approximation appears sound based on the executed sensitivity analysis in sections 5 and 6 of the supplemental. While a change in the chemical environment such as the presence of an adsorbed oxygen species, $*O_A$, in proximity to the active site may alter the adsorption energetics as well as scaling relation, the sensitivity analysis demonstrates that the overall results of the present study are not affected by such modifications. Therefore, in combination with $G_{11} = \Delta G_1$, we obtain:

$$G_{M}(U) = 0 \tag{66}$$
$$G_{M\text{-}OH}(U) = \Delta G_1 - 1 \times e \times U \tag{67}$$
$$G_{M\text{-}O}(U) = 3 \times \Delta G_1 - 2 \times e \times U \tag{68}$$
$$G_{M\text{-}OO}(U) = 4 \times \Delta G_1 - 3 \times e \times U \tag{69}$$
$$G_{M+O2}(U) = + 4.92\ \text{eV} - 4 \times e \times U \tag{70}$$

Based on the intermediate states' energetics, the free-energy spans for the bifunctional mechanism can be defined to derive $G_{max}(U)$:

$$G_{max}(U) = \max\{G_{M\text{-}OH}(U) - G_{M}(U); G_{M\text{-}O}(U) - G_{M}(U); G_{M\text{-}OO}(U) - G_{M}(U); G_{M\text{-}O}(U) - G_{M\text{-}OH}(U); G_{M\text{-}OO}(U) - G_{M\text{-}OH}(U); G_{M+O2}(U) - G_{M\text{-}OH}(U); G_{M\text{-}OO}(U) - G_{M\text{-}O}(U); G_{M+O2}(U) - G_{M\text{-}O}(U); G_{M+O2}(U) - G_{M\text{-}OO}(U)\} \tag{71}$$

The bifunctional-Walden mechanism reads:

$$\text{M-OH} + *O_A \rightarrow \text{M-O} + *O_A + H^+ + e^- \quad \Delta G_{13} \tag{72}$$
$$\text{M-O} + *O_A + H_2O \rightarrow \text{M-OO} + *OH_A + H^+ + e^- \quad \Delta G_{14} \tag{73}$$
$$\text{M-OO} + *OH_A \rightarrow \text{M-OO} + *O_A + H^+ + e^- \quad \Delta G_{15} \tag{74}$$
$$\text{M-OO} + *O_A + H_2O \rightarrow \text{M-OH} + *O_A + O_{2(g)} + H^+ + e^- \quad \Delta G_{16} \tag{75}$$

The free energies of the reaction intermediates *OH, *O, and *OO in dependence of the applied electrode potential are given by equations (76) – (80):

$$G_{M\text{-}OH}(U) = 0 \tag{76}$$
$$G_{M\text{-}O}(U) = \Delta G_{13} - 1 \times e \times U \tag{77}$$
$$G_{M\text{-}OO+*OH}(U) = \Delta G_{13} + \Delta G_{14} - 2 \times e \times U \tag{78}$$

$$G_{M\text{-}OO+{}^*O}(U) = \Delta G_{13} + \Delta G_{14} + \Delta G_{15} \qquad - 3 \times e \times U \qquad (79)$$

$$G_{M\text{-}OH+O2}(U) = \qquad + 4.92\ \text{eV} \qquad - 4 \times e \times U \qquad (80)$$

Due to $\Delta G_{13} = \Delta G_2$ and $\Delta G_{14} = \Delta G_1$, the energetics of the mononuclear-Walden mechanism can be related to the scaling relations of equations (34) and (35). Additionally, it can be shown that $\Delta G_{15} = \Delta G_2$, and thus we obtain:

$$G_{M\text{-}OH}(U) = 0 \qquad (81)$$

$$G_{M\text{-}O}(U) = \quad 2 \times \Delta G_1 \qquad - 1 \times e \times U \qquad (82)$$

$$G_{M\text{-}OO+{}^*OH}(U) = 3 \times \Delta G_1 \qquad - 2 \times e \times U \qquad (83)$$

$$G_{M\text{-}OO+{}^*O}(U) = 5 \times \Delta G_1 \qquad - 3 \times e \times U \qquad (84)$$

$$G_{M\text{-}OH+O2}(U) = \qquad + 4.92\ \text{eV} \qquad - 4 \times e \times U \qquad (85)$$

Based on the intermediate states' energetics, the free-energy spans for the bifunctional-Walden mechanism can be defined to derive $G_{max}(U)$:

$$G_{max}(U) = \max\{G_{M\text{-}O}(U) - G_{M\text{-}OH}(U); G_{M\text{-}OO+{}^*OH}(U) - G_{M\text{-}OH}(U); G_{M\text{-}OO+{}^*O}(U) - G_{M\text{-}OH}(U); G_{M\text{-}OO+{}^*OH}(U) - G_{M\text{-}O}(U); G_{M\text{-}OO+{}^*O}(U) - G_{M\text{-}O}(U); G_{M\text{-}OH+O2}(U) - G_{M\text{-}O}(U); G_{M\text{-}OO+{}^*O}(U) - G_{M\text{-}OO+{}^*OH}(U); G_{M\text{-}OH+O2}(U) - G_{M\text{-}O^*+{}^*OH}(U); G_{M\text{-}OH+O2}(U) - G_{M\text{-}OO+{}^*O}(U)\} \qquad (86)$$

Subsequently, the energetics of the bifunctional and bifunctional-Walden mechanisms is compiled in a single activity volcano plot based on the above modeling approach to determine the energetically favored pathway in dependence of the descriptor ΔG_1, depicted in **Figure S9**.

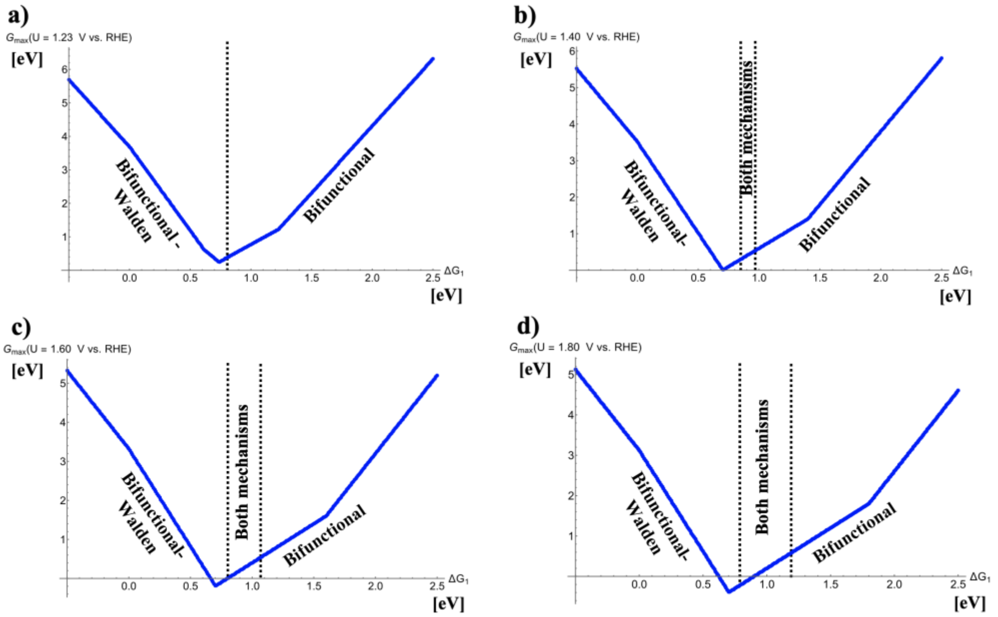

Figure S9. Potential-dependent volcano plots for the bifunctional and the bifunctional-Walden pathways of the oxygen evolution reaction at **a)** U = 1.23 V *vs*. RHE, **b)** U = 1.40 V *vs*. RHE, **c)** U = 1.60 V *vs*. RHE, and **d)** U = 1.80 V *vs*. RHE. The energetically favored mechanisms in the approximation of $G_{max}(U)$ as a potential-dependent activity measure are indicated in dependence of the adsorption free energy of the *OH intermediate, ΔG_1. To derive the volcano curves, the following scaling relations are considered: $\Delta G_2 + \Delta G_3 = 3.20$ eV and $\Delta G_2 = 2 \times \Delta G_1$.

Figure S9 illustrates that for low overpotentials, the bifunctional and bifunctional-Walden pathways are favored at the right and left volcano legs including apex, respectively. Even if both mechanisms compete with increasing overpotential on the right-hand side of the volcano, the apex of the OER volcano is governed by the bifunctional-Walden description rather than the bifunctional mechanism. This finding pinpoints the importance of the bifunctional-Walden pathway for the theoretical description of highly active OER catalysts.

The following free-energy spans limit the OER volcano plot of **Figure S9** at U = 1.23 V *vs*. RHE:

a) – 0.5 eV < ΔG_1 < 0.62 eV (bifunctional-Walden mechanism): $G_{M\text{-}OH+O2}(U) - G_{M\text{-}OO+*O}(U)$
b) 0.62 eV < ΔG_1 < 0.74 eV (bifunctional-Walden mechanism): $G_{M\text{-}OH+O2}(U) - G_{M\text{-}OO}(U)$
c) 0.74 eV < ΔG_1 < 0.81 eV (bifunctional-Walden mechanism): $G_{M\text{-}OO+*O}(U) - G_{M\text{-}OO}(U)$
d) 0.81 eV < ΔG_1 < 1.23 eV (bifunctional mechanism): $G_{M\text{-}O}(U) - G_{M\text{-}OH}(U)$
e) 1.23 eV < ΔG_1 < 2.50 eV (bifunctional mechanism): $G_{M\text{-}OO}(U) - G_{M}(U)$

8 Precondition of *OH surface groups

In section 2 of the main text, it is discussed that the occurrence of Walden inversion steps relies on the precondition that *OH groups are available under OER conditions. This finding is backed up by equations (42) – (45) as well as (72) – (75) since the catalytic cycle commences from the *OH adsorbate rather than from the unoccupied metal site, M, as in the conventional description (see equations (1) – (4)).

There are different levels of sophistication to determine whether *OH surface groups are available for the OER catalysis. Coverage of intermediate species under steady-state reaction conditions is determined by the reaction kinetics rather than by the thermodynamics **[18]**. Therefore, it is a viable measure to identify whether the formation of the *OH adsorbate refers to the rate-determining step (RDS) under OER conditions. Following reference **[19]** based on a generalized microkinetic model, *OH formation is never met with the RDS under typical OER conditions. Therefore, it can be concluded that the precondition of having *OH adsorbates on the surface is likely not violated.

The main issue of discussing the RDS refers to the fact that most theoretical studies in the density functional theory (DFT) approximation discuss the potential-determining step (PDS) **[20]** based on the reaction energetics of the elementary steps (thermodynamics) rather than the RDS since the calculation of transition states by conventional canonical (constant-charge) approaches is still in its infancy and not fully mature yet **[21,22]**. Therefore, for a dedicated discussion of the coverage of intermediate species for catalysts within a class of materials, it is rather needed to inspect the reaction energetics in terms of free energies, G, following the popular computational hydrogen electrode approach **[23]**.

A straightforward evaluation scheme refers to inspect whether *OH formation is reconciled with the PDS (cf. equation (6)) since if the generation of *OH surface groups is potential determining, this may cause a kinetic limitation when making use of the tacit assumption that PDS = RDS is met **[11,20]**. It should be noted though that the presumption of identical potential- and rate-determining steps can be violated, particularly for low overpotentials **[12]**. Therefore, one needs

to consider this evaluation scheme with a grain of salt. Following the conventional OER volcano (cf. **Figure S1**) based on linear scaling relationships between the *OH, *O, and *OOH intermediates **[2]**, *OH formation does not refer to the PDS. Hence, it can be concluded that the formation of the *OH adsorbate is not a limiting factor, and thus, the precondition of *OH surface groups under OER conditions is likely met.

In reference **[24]**, Calle-Vallejo and coworkers present a detailed analysis on the PDS for a large data set of materials ranging from transition-metal oxides, metal oxides, perovskites, porphyrins, and functionalized graphitic materials by analyzing the largest free-energy change according to equation (6). In contrast to the conventional OER volcano of **Figure S1**, they report that the formation of the *OH adsorbate constitutes the PDS in about 12 % of all cases. This is the motivation to inspect the precondition of *OH surface groups under OER conditions in more detail.

While the determination of the actual surface coverage is far beyond the scope of the present manuscript and not possible by simple thermodynamic considerations in terms of analyzing the free-energy changes of intermediate species, ΔG_j, we use the following criterion to inspect whether *OH surface groups are available under OER conditions: if the condition $\Delta G_1 < 1.50$ eV (cf. equation (1)) is fulfilled, the surface of the electrocatalyst consists of *OH groups, and thus, mechanistic steps including the Walden inversion can occur. We discuss this criterion for several classes of materials based on the data in references **[24-27]**.

Table S1. Analysis of the free-energy change ΔG_1 referring to *OH formation for various material classes, and assessment of the electrocatalytic activity in terms of the thermodynamic overpotential (cf. equation (6)) in case that $\Delta G_1 > 1.50$ eV is met.

**Material class**	**$\Delta G_1 < 1.50$ eV**	**$\Delta G_1 > 1.50$ eV**	**$\eta_{TD} < 0.60$ eV (if $\Delta G_1 > 1.50$ eV)**
Porphyrins (# 36)	28	8	0
Perovskites (# 24)	13	11	3
Metal oxides (# 10)	8	2	0
Functional graphitic materials (# 13)	7	6	0
Doped TiO_2 (# 56)	34	22	2
Transition-metal oxides (# 7)	6	1	0
Oxyhydroxides (# 31)	31	0	0

Table S1 reveals that in more than 70% of all cases, the criterion $\Delta G_1 < 1.50$ eV is met, and thus, surface *OH groups are available for the OER catalysis including Walden inversion steps. In less than 30% of all cases, the presence of surface *OH groups is questionable due to the thermodynamic restraint of *OH formation. For this specific situation, it is further analyzed whether these materials are highly active, adopting $\eta_{TD} < 0.60$ eV as a threshold for highly active catalysts **[11,25]**. It turns out that in 5 out of 177 cases, a highly active catalyst ($\eta_{TD} < 0.60$ eV) reveals $\Delta G_1 > 1.50$ eV, and thus, the occurrence of Walden inversion steps is debatable. This refers to less than 3 % of the entirely considered material space, and thus, the main conclusions of this manuscript relating to the importance of Walden inversion steps for highly active catalytic materials at the apex of the OER volcano plot does not break down.

References

[1] Rossmeisl, J.; Logadottir, A.; Nørskov, J. K. Electrolysis of Water on (Oxidized) Metal Surfaces. Chem. Phys. 2005, 319, 178-184.

[2] Man, I. C.; Su, H.-Y.; Calle-Vallejo, F.; Hansen, H. A.; Martinez, J. I.; Inoglu, N. G.; Kitchin, J.; Jaramillo, T. F.; Nørskov, J. K.; Rossmeisl, J. Universality in Oxygen Evolution Electrocatalysis on Oxide Surfaces. ChemCatChem 2011, 3, 1159-1165.

[3] Koper, M. T. M. Analysis of electrocatalytic reaction schemes: Distinction between rate-determining and potential-determining steps. J. Solid State Electrochem. 2013, 17, 339-344.

[4] Nørskov, J. K.; Bligaard, T.; Rossmeisl, J.; Christensen, C. H. Towards the computational design of solid catalysts. Nat. Chem. 2009, 1, 37-46.

[5] Exner, K. S. On the mechanistic complexity of oxygen evolution: potential-dependent switching of the mechanism at the volcano apex. Mater. Horiz. 2023, 10, 2086-2095.

[6] Busch, M.; Ahlberg, E.; Panas, I. Electrocatalytic oxygen evolution from water on a Mn(iii–v) dimer model catalyst—A DFT perspective. Phys. Chem. Chem. Phys. 2011, 13, 15069-15076.

[7] Ping, Y.; Nielsen, R. J.; Goddard, W. A. The Reaction Mechanism with Free Energy Barriers at Constant Potentials for the Oxygen Evolution Reaction at the IrO_2(110) Surface. J. Am. Chem. Soc. 2017, 139, 149-155.

[8] Ha, M.-A.; Larsen, R. E. Multiple Reaction Pathways for the Oxygen Evolution Reaction May Contribute to IrO_2 (110)'s High Activity. J. Electrochem. Soc. 2021, 168, 024506.

[9] Binninger, T.; Doublet, M. L. The Ir–OOOO–Ir transition state and the mechanism of the oxygen evolution reaction on IrO_2(110). Energy Environ. Sci. 2022, 15, 2519-2528.

[10] Exner, K. S. Toward data- and mechanistic-driven volcano plots in electrocatalysis. Electrochem. Sci. Adv. 2023, DOI: 10.1002/elsa.202200014.

[11] Exner, K. S. A Universal Descriptor for the Screening of Electrode Materials for Multiple-Electron Processes: Beyond the Thermodynamic Overpotential, ACS Catal. 2020, 10, 12607-12617.

[12] Razzaq, S.; Exner, K. S. Materials Screening by the Descriptor $G_{max}(\eta)$: The Free-Energy Span Model in Electrocatalysis. ACS Catal. 2023, 13, 1740-1758.

[13] Exner, K. S. Importance of the volcano slope to comprehend activity and selectivity trends in electrocatalysis. Curr. Opin. Electrochem. 2023, 39, 101284.

[14] Exner, K. S. Steering Selectivity in the Four-Electron and Two-Electron Oxygen-Reduction Reactions: On the Importance of the Volcano Slope. ACS Phys. Chem. Au 2023, 3, 190-198.

[15] Viswanathan, V.; Hansen, H. A. Unifying solution and surface electrochemistry: limitations and opportunities in surface electrocatalysis. Top. Catal. 2014, 57, 215-221.

[16] Calle-Vallejo, F.; Krabbe, A.; Garcia-Lastra, J. M. How Covalence Breaks Adsorption-Energy Scaling Relations and Solvation Restores Them. Chem. Sci. 2017, 8, 124-130.

[17] Exner, K. S. Design Criteria for Oxygen Evolution Electrocatalysts from First Principles: Introduction of a Unifying Material-Screening Approach. ACS Appl. Energy Mater. 2019, 2, 7991-8001.

[18] Huang, J.; Zhu, X.; Eikerling, M. The rate-determining term of electrocatalytic reactions with first-order kinetics. Electrochim. Acta 2021, 393, 139019.

[19] Exner, K. S. Universality in Oxygen Evolution Electrocatalysis: High-Throughput Screening and a Priori Determination of the Rate-Determining Reaction Step. ChemCatChem 2020, 12, 2000-2003.

[20] Koper, M. T. M. Analysis of electrocatalytic reaction schemes: Distinction between rate-determining and potential-determining steps. J. Solid State Electrochem. 2013, 17, 339-344.

[21] Chan, K.; Nørskov, J.K. Potential dependence of electrochemical barriers from ab initio calculations. J. Phys. Chem. Lett. 2016, 7, 1686-1690.

[22] Groß, A. Grand-canonical approaches to understand structures and processes at electrochemical interfaces from an atomistic perspective. Curr. Opin. Electrochem. 2021, 27, 100684.

[23] Nørskov, J. K.; Rossmeisl, J.; Logadottir, A.; Lindqvist, L.; Kitchin, J. R.; Bligaard, T.; Jonsson, H. J. Origin of the Overpotential for Oxygen Reduction at a Fuel-Cell Cathode. J. Phys. Chem. B 2004, 108, 17886-17892.

[24] Pique, O.; Illas, F.; Calle-Vallejo, F. Designing water splitting catalysts using rules of thumb: Advantages, dangers and alternatives. Phys. Chem. Chem. Phys. 2020, 22, 6797-6803.

[25] Govindarajan, N.; Koper, M. T. M.; Meijer, E. J.; Calle-Vallejo, F. Outlining the Scaling-Based and Scaling-Free Optimization of Electrocatalysts. ACS Catal. 2019, 9, 4218-4225.

[26] Sumaria, V.; Krishnamurthy, D.; Viswanathan, V. Quantifying Confidence in DFT Predicted Surface Pourbaix Diagrams and Associated Reaction Pathways for Chlorine Evolution. ACS Catal. 2018, 8, 9034-9042.

[27] Hu, Q.; Xue, Y.; Kang, J.; Scivetti, I.; Teobaldi, G.; Selloni, A.; Guo, L.; Liu, L.-M. Structure and Oxygen Evolution Activity of β-NiOOH: Where Are the Protons? ACS Catal. 2022, 12, 295-304.
